# Ectopic enhancer–enhancer interactions as causal forces driving RNA‐directed DNA methylation in gene regulatory regions

**DOI:** 10.1111/pbi.14435

**Published:** 2024-07-17

**Authors:** Yazhou Yang, Jia Liu, Stacy D. Singer, Guohua Yan, Dennis R. Bennet, Yue Liu, Jean‐Michel Hily, Weirong Xu, Yingzhen Yang, Xiping Wang, Gan‐Yuan Zhong, Zhongchi Liu, Yong‐Qiang Charles An, Huawei Liu, Zongrang Liu

**Affiliations:** ^1^ College of Horticulture Northwest A&F University Yangling China; ^2^ College of Landscape, Architecture and Life science/Institute of Special Plants Chongqing University of Arts and Sciences Yongchuan Chongqing China; ^3^ Agriculture and Agri‐Food Canada, Lethbridge Research and Development Centre Lethbridge Alberta Canada; ^4^ The Institute of Forestry and Pomology, Beijing Academy of Agriculture and Forestry Sciences Beijing China; ^5^ USDA‐ARS Appalachian Fruit Research Station Kearneysville West Virginia USA; ^6^ College of Horticulture Qingdao Agricultural University Qingdao China; ^7^ Institut Français de la Vigne et du Vin (IFV) Le Grau du Roi France; ^8^ School of Food & Wine Ningxia University Yinchuan Ningxia China; ^9^ USDA‐ARS, Grape Genetic Research Unit Geneva New York USA; ^10^ Department of Cell Biology and Molecular Genetics University of Maryland College Park Maryland USA; ^11^ USDA‐ARS, Plant Genetics Research Unit, Donald Danforth Plant Science Center St Louis Missouri USA; ^12^ Xinjiang Institute of Ecology and Geography, Chinese Academy of Sciences Urumqi China

**Keywords:** *CRE–CRE* interactions, ncRNA transcription, RdDM, *de novo* methylation, transcriptional silencing, mutant flowers

## Abstract

*Cis*‐regulatory elements (*CREs*) are integral to the spatiotemporal and quantitative expression dynamics of target genes, thus directly influencing phenotypic variation and evolution. However, many of these *CREs* become highly susceptible to transcriptional silencing when in a transgenic state, particularly when organised as tandem repeats. We investigated the mechanism of this phenomenon and found that three of the six selected flower‐specific *CREs* were prone to transcriptional silencing when in a transgenic context. We determined that this silencing was caused by the ectopic expression of non‐coding RNAs (ncRNAs), which were processed into 24‐nt small interfering RNAs (siRNAs) that drove RNA‐directed DNA methylation (RdDM). Detailed analyses revealed that aberrant ncRNA transcription within the *AGAMOUS* enhancer (*AGe*) in a transgenic context was significantly enhanced by an adjacent *CaMV35S* enhancer (*35Se*). This particular enhancer is known to mis‐activate the regulatory activities of various *CREs,* including the *AGe*. Furthermore, an insertion of *35Se* approximately 3.5 kb upstream of the *AGe* in its genomic locus also resulted in the ectopic induction of ncRNA/siRNA production and *de novo* methylation specifically in the *AGe*, but not other regions, as well as the production of mutant flowers. This confirmed that interactions between the *35Se* and *AGe* can induce RdDM activity in both genomic and transgenic states. These findings highlight a novel epigenetic role for *CRE–CRE* interactions in plants, shedding light on the underlying forces driving hypermethylation in transgenes, duplicate genes/enhancers, and repetitive transposons, in which interactions between *CREs* are inevitable.

## Introduction

*Cis*‐regulatory elements (*CREs*) located in the promoters and enhancers are known to regulate spatiotemporal and quantitative expression dynamics of target genes in response to internal or external cues (Marand *et al*., 2017; Weber *et al*., 2016). This regulation contributes to phenotypic variation and functional evolution. Generally, *CREs* remain epigenetically stable in their native genomic state. However, under certain circumstances such as exposure to environmental, physiological or genomic stresses in plants or during aging in mammals, some *CREs* can become vulnerable to cytosine methylation and transcriptional silencing, leading to phenotypic alterations in plants (Manning *et al*., 2006; Quadrana *et al*., 2014; Soppe *et al*., 2000; Tan *et al*., 2018) and disease symptoms in humans (Jin and Liu, 2018; Laird, 2003; Robertson, 2005; Yanovsky‐Dagan *et al*., 2015). This methylation vulnerability becomes even more pronounced when *CREs* are in a transgenic state (Elmayan and Vaucheret, 1996; Flavell, 1994; Henikoff and Comai, 1998; Kumar and Fladung, 2000; Meyer and Heidmann, 1994; Sasaki *et al*., 2014), which hints at the possibility that the genomic context or transgenic state itself may play a role in susceptibility.

Although methylation pathways have been well‐characterised, particularly in plants (Law and Jacobsen, 2010; Matzke and Mosher, 2014; Zhang *et al*., 2018), the precise mechanisms behind transgene state‐dependent *de novo* methylation remain unclear. It has been demonstrated that canonical RdDM is largely responsible for *de novo* methylation and is required for methylation in euchromatic regions, short transposons and around the edges of long heterochromatic transposons (Chan *et al*., 2006; Law and Jacobsen, 2010; Matzke and Mosher, 2014; Zemach *et al*., 2013; Zhang *et al*., 2018). Canonical RdDM is initiated by 24‐nt siRNAs derived from non‐coding RNAs transcribed by a plant‐specific nuclear RNA polymerase IV (Pol IV) (Blevins *et al*., 2015; Li *et al*., 2015; Zhai *et al*., 2015). These siRNAs, which are bound to an ARGONAUTE (AGO) effector complex containing AGO4 and AGO6, then interact with long non‐coding RNAs (lncRNAs) transcribed by a plant‐specific nuclear RNA Pol V. The lncRNAs act as a scaffold, recruiting multiple protein factors, including REARRANGED METHYLTRANSFERASE 2 (DRM2), which is a homologue of the mammalian DNMT3 methyltransferase (Zhong *et al*., 2014). The lncRNA‐associated complex mediates the methylation of cytosines, guided by the 24‐nt siRNAs (Law and Jacobsen, 2010; Matzke and Mosher, 2014; Zhang *et al*., 2018). The availability of ncRNAs is the key factor determining RdDM activity and *de novo* methylation in *CREs*, which is largely dependent on ncRNA transcription by Pol IV and could be influenced by inherent genomic context, Pol IV activity and *CRE* accessibility, as well as responsiveness to internal and external signals or stresses.

Interestingly, transgenic enhancers/promoters that are tandemly oriented have been found to be particularly vulnerable to DNA methylation/silencing (Bender and Fink, 1995; Elmayan and Vaucheret, 1996; Flavell, 1994; Henikoff and Comai, 1998; Kumar and Fladung, 2000; Meyer and Heidmann, 1994; Sasaki *et al*., 2014). This configuration‐dependent methylation susceptibility is similar to the methylation propensity seen in regions of the genome rich in repetitive transposon elements (Martienssen, 2003), suggesting that this configuration or unknown associated factors may mediate the enhancement of RdDM activity. This theory is supported by previous studies of paramutation, which is an epigenetic phenomenon observed at specific loci in maize (Arteaga‐Vazquez and Chandler, 2010; Hollick, 2017) and occurs when paramutagenic (silent) and paramutable (non‐silent but responsive to the silencing state of the paramutagenic allele) alleles communicate with each other, leading to meiotically heritable transcriptional silencing of paramutable alleles (Arteaga‐Vazquez and Chandler, 2010). Intriguingly, for paramutation to occur, alleles must bear tandemly repetitive enhancers (Arteaga‐Vazquez and Chandler, 2010; Hollick, 2017), and the strength of paramutation is correlated with the number of enhancer/*CRE* copies (Kermicle *et al*., 1995; Panavas *et al*., 1999; Stam *et al*., 2002). Furthermore, paramutation is associated with the transcription of ncRNAs, siRNA production, and hypermethylation, and requires protein factors that are homologous to RdDM components found in *Arabidopsis* (Alleman *et al*., 2006; Arteaga‐Vazquez *et al*., 2010; Belele *et al*., 2013; Haring *et al*., 2010; Stam *et al*., 2002). Similarly, in *Arabidopsis*, tandem repeats in the *FLOWERING WAGENINGEN* (*FWA*) promoter are highly vulnerable to hypermethylation (Kinoshita *et al*., 2004, 2006; Soppe *et al*., 2000), and this vulnerability also requires the RdDM pathway (Chan *et al*., 2004). These forms of tandem repeat‐dependent silencing suggest that enhancer or *CRE* duplication could emanate an unknown regulatory force that drives ncRNA transcription and ensuing RdDM activity.

Previous studies have demonstrated that *CREs* in duplicated genes within a genome, or within a transgene, interact strongly, leading to a synergistic increase in expression levels beyond any correlation to copy number (Kay *et al*., 1987; Loehlin *et al*., 2022; Loehlin and Carroll, 2016). Such interactions are unavoidable in the case of duplicated enhancers, tandemly oriented transgenes and repetitive genomic regions, where multiple *CREs* are present. These interactions also occur between exogenously inserted *CREs* and adjacent *CREs* in plant genomes, as exemplified by reinserted transposon *CREs* or transgenic *CREs* that activate, or are activated by, adjacent *CREs* in genomes (Chuong *et al*., 2017; Gudynaite‐Savitch *et al*., 2009; Hayashi and Yoshida, 2009; Hirsch and Springer, 2017; Hollister and Gaut, 2009; Kashkush *et al*., 2003). While interactions between *CREs*, which have been widely used for activation tagging analysis of gene function (Weigel *et al*., 2000) and for enhancer trapping analysis of a *CRE's* tissue‐specificity (Acosta‐García *et al*., 2004), occur across different types and groups of *CREs* (Chuong *et al*., 2017; Gudynaite‐Savitch *et al*., 2009; Hayashi and Yoshida, 2009; Hollister and Gaut, 2009; Kashkush *et al*., 2003; Wen *et al*., 2014), they are not uniform, with some *CREs* displaying stronger responses than others and others exhibiting resistance to interactions (Gudynaite‐Savitch *et al*., 2009; Liu *et al*., 2008; Wen *et al*., 2014). However, as of yet, it remains unclear whether these interactions act as a fundamental mechanism triggering the ectopic transcription of ncRNAs and RdDM in transgenic *CREs*, duplicated enhancers/genes and repetitive transposon‐rich regions.

In this study, we conducted extensive analyses to explore how methylation vulnerability in transgenic *CREs* is activated, what causes it and whether different *CRE*‐containing transgenes have similar susceptibilities. Our findings indicated that three of the six selected flower‐specific transgenic *CREs* were susceptible to transcriptional silencing, which was dependent on the ectopic production of ncRNAs from within the transgenic *CREs*. Furthermore, we demonstrated that *CRE*–*CRE* interactions can activate ncRNA transcription/siRNA production and RdDM activity, supporting the hypothesis that *CRE*–*CRE* interactions are a causal force driving RdDM in interacting *CREs*.

## Results

### Three of six *Arabidopsis* flower‐specific transgenic *CREs* are highly prone to RdDM/silencing

Previous studies have demonstrated that silencing the promoters/*CREs* of genes controlling flowering time and floral structure can cause mutant phenotypes similar to those resulting from *loss‐of‐function* mutations (Jacobsen and Meyerowitz, 1997; Soppe *et al*., 2000). We leveraged this phenotype‐based approach to investigate the methylation/silencing vulnerability of six *CREs* associated with flower‐development genes whose loss‐of‐function mutations give rise to distinct mutant flower phenotypes (Bowman *et al*., 1989, 1992; Ferrandiz *et al*., 2000; Gu *et al*., 1998; Jack *et al*., 1992; Jacobsen and Meyerowitz, 1997; Weigel *et al*., 1992). These included the promoters of *APELETA1* (*AP1p*), *APELETA3* (*AP3p*), *LEAFY* (*LFYp*), *AGAMOUS‐LIKE 8* (*AGL8p*) and *SUPERMAN* (*SUPp*), and the enhancer located in the second intron of *AGAMOUS* (*AGe*). The isolated promoters/enhancers were then re‐introduced into *Arabidopsis* as transgenes without any modification or other strong *CREs*/promoter present in an immediately adjacent position in the binary vector (Figure S1a).

**Figure 1:**
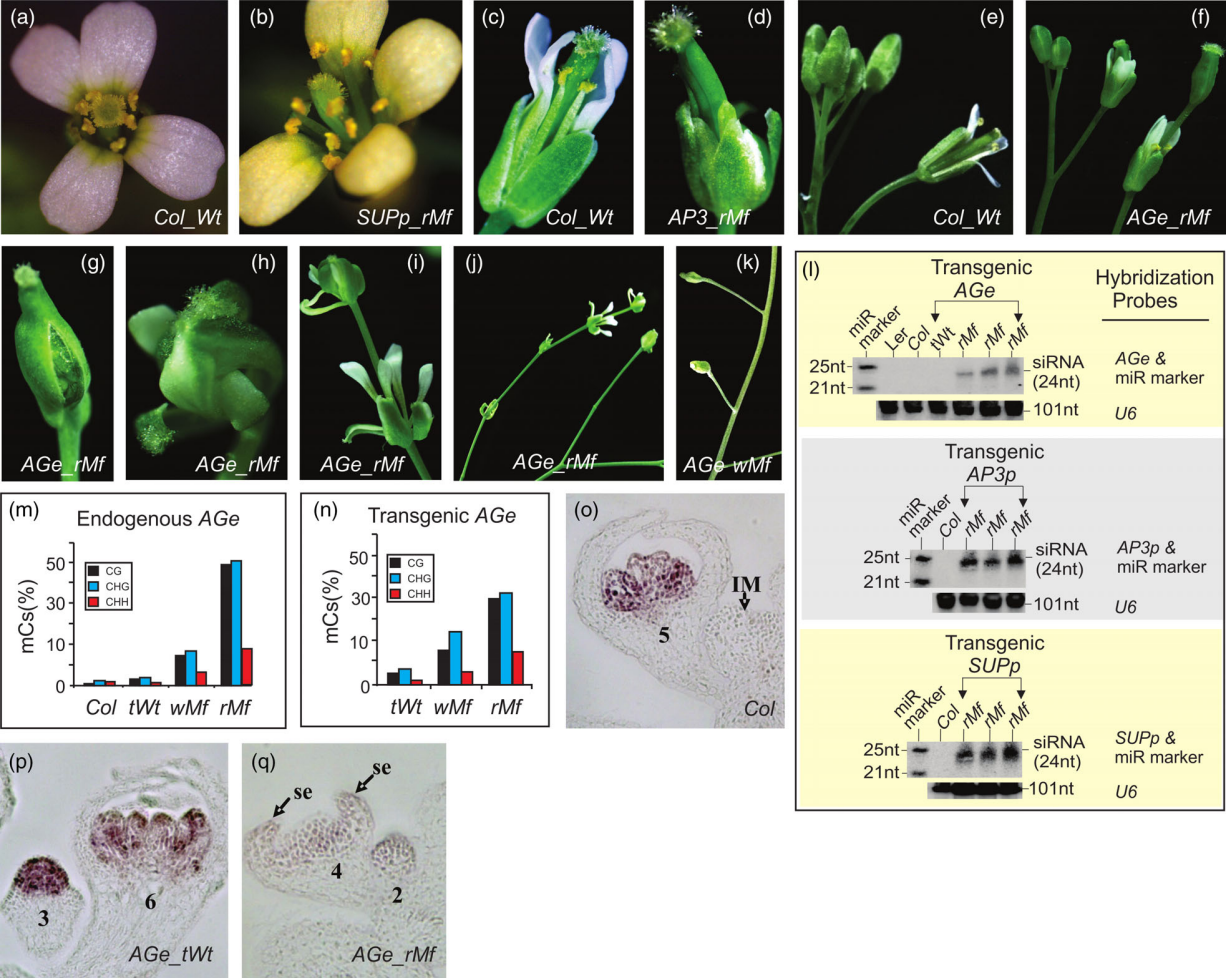
Transgenic silencing of flower‐specific enhancer/promoters. (a, c, e) *Wt* (*Col*) flower. (b, d, f–k) Mutant flowers in *SUPp* (b)‐, *AP3p* (d)‐ and *AGe* (f–k)‐transformed lines. (f–k) regular (*rMf*; f–j) and weak (*wMf*; k) mutant flowers (*Mfs*) of *AGe*‐transformed lines. (l) sRNA gel blot for the detection of 24‐nt siRNA production in three transgenic line with regular mutant flowers (*rMfs*) induced by *AGe* (top panel), *AP3p* (middle panel) and *SUPp* (bottom panel) transgenes, respectively. An RNA loading control was assessed using the same filters/blots stripped and co‐hybridised with the labelled *Arabidopsis RNU6‐1* (*U6*), miR319 and miRNA marker. A detailed description of the gel blot results can be found in Figure S1c–h. The sizes of the co‐hybridised miRNA marker are displayed on the left, while 24‐nt siRNAs and 101‐nt *U6* RNAs are shown on the right. miR marker – miRNA marker. *Ler – Arabidopsis Landsberger* ecotype. *Col* – *Arabidopsis Columbia* ecotype. *tWt* – transgenic line with *Wt* flowers. (m, n) PCR bisulphite sequencing analysis of cytosine methylation in CG, CHG and CHH contexts (H = A, T, or C) in the 1726‐bp endogenous (m) and transgenic (n) *AGe* regions of *Wt* flowers, and *AGe* transgenic *Wt* (*tWt*), *wMf* and *rMf* flowers, respectively. %mCs – The frequency of methylated cytosines (mCs) in each position out of the total number of cytosines. (o–q) *In situ* hybridisation analysis of *AG* expression in *Wt* (o), *AGe tWt* (p) and *rMf* (q) flowers, respectively. Specific floral stages (e.g. 2, 3, 4, 5, 6) defined previously (Smyth *et al*., 1990) are indicated. IM – inflorescence meristem; se – sepal.

Transgenic analyses demonstrated that the *AGe, AP3p* and *SUPp* transgenes induced distinct mutant flower (*Mf*) phenotypes in approximately 42%, 44% and 28% of T_1_ transgenic populations, respectively (Table 1). *SUPp* mutant flowers displayed 7–12 stamens instead of the standard 6 in *Wt* flowers (Figure 1a,b), similar to loss‐of‐function or epi *sup* mutant flowers (Bowman *et al*., 1992; Jacobsen and Meyerowitz, 1997). *AP3p* mutant flowers exhibited an absence of petals and stamens (Figure 1c,d), similar to loss‐of‐function *ap3* mutant flowers (Jack *et al*., 1992). *AGe* lines, on the other hand, exhibited two *Mf* phenotypes. Less than 10% of *AGe* mutant lines displayed either enlarged carpels that failed to develop further or normal appearing but sterile flowers (Figures 1k and S1b), which are collectively called the weak mutant flower (*wMf*) phenotype. The majority of the abnormal *AGe* lines, however, possessed an enlarged carpel with an elongated pedicel that subsequently gave rise to secondary flowers (Figure 1e–j). This process repeated itself, forming a chain‐like flower or regular mutant flower (*rMf*) phenotype (Figures 1j and S1b), which is rarely seen in loss‐of‐function *ag* mutants (Bowman *et al*., 1989), but is observed in *AG* RNAi plants (Mizukami and Ma, 1995) and occasionally in *Met1* RNAi plants (Jacobsen *et al*., 2000).

**Table 1:** Evaluation of the silencing susceptibility of flower‐specific *CREs*

Plasmid	DNA fragment	No. total transgenic lines	No. mutant flower lines	% mutant flower lines
*pAGe/JM40*	*AG* enhancer (*AGe*)	88	37	42
*pAGL8p*	*AGL8* promoter (*AGL8p*)	84	0	0
*pAP1p*	*AP1* promoter (*AP1p*)	89	0	0
*pAP3p*	*AP3* promoter (*AP3p*)	78	34	44
*pLFYp*	*LFY* promoter (*LFYp*)	98	0	0
*pSUPp*	*SUPPERMAN* promoter (*SUPp*)	101	28	28

To ascertain whether these mutant flower phenotypes resulted from RdDM‐mediated transcriptional silencing, we performed sRNA gel blot analysis and detected the production of 24‐nt siRNAs in *AGe*, *AP3p* and *SUPp* mutant flowers (Figures 1l and S1c–h). Since 24‐nt siRNAs serve as a causal molecule for canonical RdDM (Law and Jacobsen, 2010), we postulated that these three transgenic *CREs* co‐silenced their endogenous counterparts via RdDM, leading to the respective mutant phenotypes. To gain further insight into this phenomenon, we focused on the *AGe*‐induced silencing event.

Initially, a series of deletions were created that identified a 1726‐bp region located at the *AGe*'s 3′ terminus (e.g. *JM43*), which is bound by a dozen transcription factors that are critical for flower‐specific *AG* expression (Figures S1i and S2; Busch *et al*., 1999; Lohmann *et al*., 2001), as the sole region responsible for the observed *Mf* phenotype (Figure S1i). We then carried out PCR bisulphite sequencing and demonstrated that both transgenic and endogenous copies of the 1726‐bp *AGe* were hypermethylated in all CG, CHG and CHH (H = A, T, or C) contexts, with the highest levels detected in *rMfs*, followed by *wMfs*. In comparison, methylation was nearly undetectable in *Wt* (*Col*) and transgenic *Wt* (*tWt*) flowers (Figures 1m,n and S1j). *In situ* hybridisation analysis revealed that *AG* transcript abundance was drastically reduced in stamen and carpel primordia from stage 3 onward in *rMf* flowers (Figure 1q), but not in *Wt* and *tWt* flowers (Figure 1o,p), supporting the critical role of *AGe* in *AG* expression regulation.

### Transgenic *AGe* ‐induced RdDM is mediated by the canonical RdDM pathway

We next characterised the *AGe*‐RdDM pathway by introducing the same *AGe* transgene into characterised mutants of genes that act in canonical RdDM and post‐transcriptional gene silencing (PTGS) pathways. We tested 32 mutants, including single, double and triple mutants, and observed that 11 of them lost the ability to produce *Mfs* (Figure S3a,c). These genetic mutants included *RNA‐dependent RNA polymerase 2–1* (*rdr2‐1*), *nuclear RNA polymerase 1a‐1* (*nrpd1a‐1/pol iv*), *nrpd1b‐11 (pol v*), *nrpd1a‐1/nrpd1b‐11*, *nrpe5‐1* (*pol v*), *nrpdd2a‐1/nrpd2b‐1* (*pol iv*), *defective in RNA‐directed DNA methylation 1–6* (*drd1‐6*), *drm2‐2, drm1/drm2/drm3*, *dicer‐like‐2, ‐3, ‐4* (*dcl2)/dcl3/dcl4* and *ago4/ago6*, which are all required for canonical RdDM (Kanno *et al*., 2005; Law and Jacobsen, 2010; Stroud *et al*., 2013). The inability to induce *Mf* production was correlated with a loss of 24‐nt siRNA production in the flowers of all of the above‐mentioned genetic mutants, with the exception of *drd1*, where 24‐nt siRNAs were detected in one of two *Mf* lines (Figure S3b).

Interestingly, only the *dcl2/dcl3/dcl4* triple mutant lost siRNA biogenesis, DNA methylation and *Mf* production, while single mutants or combinations of two mutants did not (Figure S3a–f). This indicates that *DCL2, DCL3* and *DCL4* are functionally redundant, as loss of *DCL3* function was apparently compensated for by *DCL2*, coupled with a switch in production from 24‐nt to 22‐nt siRNAs. The *dcl2/dcl3* double mutant was also partially recouped by *DCL4*, which was associated with the predominant production of 21‐nt siRNAs, reduced methylation and the generation of *wMfs* (Figure S3c–e), suggesting that 21‐nt siRNAs are less effective than 22‐nt and 24‐nt siRNAs in terms of inducing RdDM and the *Mf* phenotype. Our findings also indicate that the three *DCLs* behave hierarchically, in line with evidence from previous studies (Bond and Baulcombe, 2015; Henderson *et al*., 2006).

### The *AGe* transgene transcribes ncRNAs from both strands in *Mfs*

**Figure 2:**
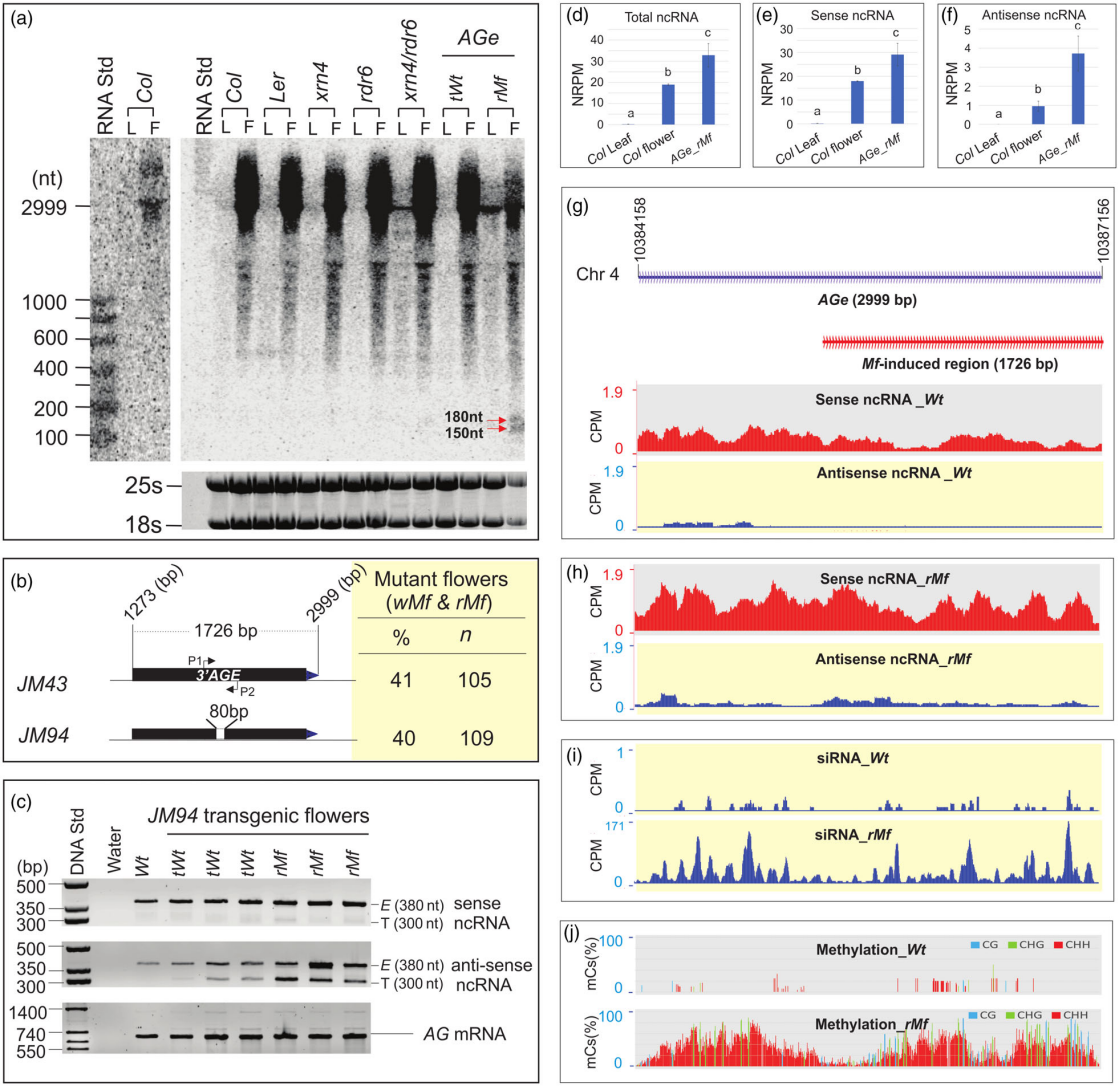
Detection of *AGe* transgene‐specific activation of ncRNA transcription and RdDM events in *rMf* flowers. (a) Regular RNA gel blot. The filter was initially hybridised with a ^32^P‐labelled *AGe* fragment (right panel), followed by probing of ^32^P‐labelled RNA marker template DNA (left panel). 25s and 18s rRNAs in the bottom panel served as loading controls (bottom panel). RNA std – RNA standard marker; L – leaf; F – flower; *Col* – *Arabidopsis Columbia* ecotype; Ler – *Arabidopsis Landsberg erecta*; *rdr6* – mutant of *RNA‐DEPENDENT RNA POLYMERASE 6 (RDR6)*; *xrn4* – mutant of *5′‐3′ exoribonuclease 4 (XRN4)*; *xrn4/rdr6* – double mutant; *tWt* – transgenic *Wt* flower; *rMf* – regular mutant flower; red arrows – indication of approximately 150‐nt and 180‐nt RNA bands. (b, c) An internal 80‐bp deletion (*JM94*) within the 3′ 1726‐bp *AGe* region in *JM43* (b), and differentiation between transgenic (300‐bp) and endogenous (380‐bp) *AGe*‐transcribed ncRNAs using strand‐specific RT‐PCR with P1/AGI‐IIF6 and P2/AGI‐IIR7 primer pairs (c). E – transcript from the endogenous *AGe*; T – transcript from the transgenic *AGe*. P1 – forward primer. P2 – reverse primer that located 380 bp downstream of P1 primer. (d–f) Strand‐specific RNA‐seq analysis of total (d), sense (e) and anti‐sense (f) ncRNAs transcribed from the *AGe* region in *Wt* leaves and flowers, and *rMf* flowers, respectively. Data were averaged from three biological replicates, with ±SD. Significant differences among tissues from different lines are indicated by lowercase letters (*P* < 0.05, ANOVA). NRPM – Normalised reads per million mapped reads in the *AGe* region. (g, h) Mapping of sense (top panel) and anti‐sense (bottom panel) ncRNA reads along the *AGe* region in *Wt* (g) and *rMf* (h) flowers, respectively. The entire 2999‐bp *AGe* region (blue) and its internal 3′ 1726‐bp *AGe* region (red) are denoted in the top panel of (g). (i) sRNA production pattern along the *AGe* region in *Wt* (top panel) and *rMf* (bottom panel) flowers. CPM – Count per million mapped reads. (j) Cytosine methylation along the *AGe* in *Wt* (top panel) and *rMf* (bottom panel) flowers, respectively. %mCs – The frequency of methylated cytosines (mCs) in each position out of the total number of cytosines.

Since siRNA precursors/ncRNAs are indispensable for canonical RdDM activity, we performed RNA gel blot analysis to determine whether the *AGe* transgene could transcribe ncRNAs in *Mfs*. A major 2999‐nt band that corresponded with the *AGe*/second intron transcript spliced from *AG* pre‐mRNA was abundant in flowers, but barely detectable in the leaves, of *Wt* (*Col*, *Ler*) plants (Figure 2a). This is consistent with the flower‐specific expression of *AG* (Busch *et al*., 1999; Yanofsky *et al*., 1990) and splicing of the *AGe*/second intron (Cheng *et al*., 2003). A multitude of smaller bands were also detected in the *AGe*/second intron region. These bands may, at least in part, reflect secondary splicing or degraded products. Interestingly, the intensity of the 2999‐nt band was drastically reduced in *rMf* flowers compared to both *Col* and *Ler Wt*, as well as *tWt* flowers, suggesting that 2999‐nt RNAs spliced from *AG* pre‐mRNAs may be converted to siRNAs in *rMfs*. Two smaller bands, roughly corresponding to 150‐ and 180‐nt ncRNA species, were also detected specifically in *rMfs*, but were almost undetectable in *Wt* and *tWt* flowers (Figure 2a). These two small RNAs were absent in *rdr6* and *xrn4* single mutants, as well as *xrn4/rdr6* double mutants (Figure 2a), which are defective in RNA degradation/metabolism (Gazzani *et al*., 2004), suggesting that 150‐ and 180‐nt ncRNAs are not produced/transcribed in *Wt* flowers. Additionally, bands roughly corresponding to 250‐ to 600‐nt RNAs also appeared more intense in *rMfs* than *Wt* flowers. Therefore, RNA abundance and diversity in *rMfs* differ from *Wt* and *tWt* flowers. We will collectively refer to these *AGe*‐derived/transcribed RNAs as ncRNAs hereafter.

To ascertain whether ncRNAs were transcribed from the endogenous or transgenic *AGe* copy, we created an 80‐bp internal deletion within the 1726‐bp 3′ *AGe* region in the *JM94* vector and found that this deletion did not compromise its ability to induce *Mf* production (Figures 2b and S1i). However, it did allow transgenic *AGe*‐transcribed ncRNAs to be discriminated from endogenous *AGe*‐derived transcripts by PCR amplification using a primer pair that is expected to yield a 380‐bp fragment in the case of endogenous ncRNAs and a 300‐bp fragment in the case of transgenic ncRNAs, respectively (Figure 2b). Strand‐specific RT‐PCR analysis revealed that in both *Wt* and transgenic flowers, the expected 380‐nt sense and anti‐sense ncRNAs generated from the native *AGe* region were amplified, with a relative abundance observed in transgenic flowers, and especially in those with the *rMf* phenotype (Figure 2c). In contrast, while the expected 300‐nt sense ncRNA derived from the *AGe* transgene was barely detectable in the flowers of any line assessed (Figure 2c), the anti‐sense ncRNA was amplified in transgenic (*tWt*, *rMf*), but not *Wt*, flowers, and was particularly pronounced in *rMfs* (Figure 2c). This suggests that the transgenic *AGe* preferentially transcribed anti‐sense ncRNAs in *Mfs*, which was further confirmed by strand‐specific RNA‐seq (ssRNA‐seq) analysis that showed a significant increase in ncRNAs from both strands, but particularly the anti‐sense strand, in *rMfs* compared to *Wt* flowers (Figure 2d–f).

We then mapped ssRNA‐seq reads to the *AGe* region to determine where ncRNAs were transcribed from. As expected, sense ncRNA reads accumulated abundantly across the entire *AGe* region in both *Wt* and *rMf* flowers (Figure 2g,h), but anti‐sense ncRNA reads mainly mapped to the 5′ region of the *AGe* of *Wt* flowers (Figure 2g). However, in *rMfs*, anti‐sense ncRNA reads accumulated across the entire *AGe* (Figure 2h), which appears to correlate with an *AGe*‐wide increase in siRNA production and DNA methylation (Figure 2i,j). Interestingly, siRNA production displayed a small discrete peak pattern along the *AGe* region, with most siRNA peaks spanning roughly 90‐ to 150‐bp regions (Figure 2i), which coincides well with the size of the 150‐nt and 180‐nt ncRNAs detected by RNA gel blot in *rMf*s (Figure 2a).

### Insertion of a *CaMV35S* enhancer ( *35Se* ) next to the *AGe* increases ncRNA and siRNA abundance, as well as *Mf* production

**Figure 3:**
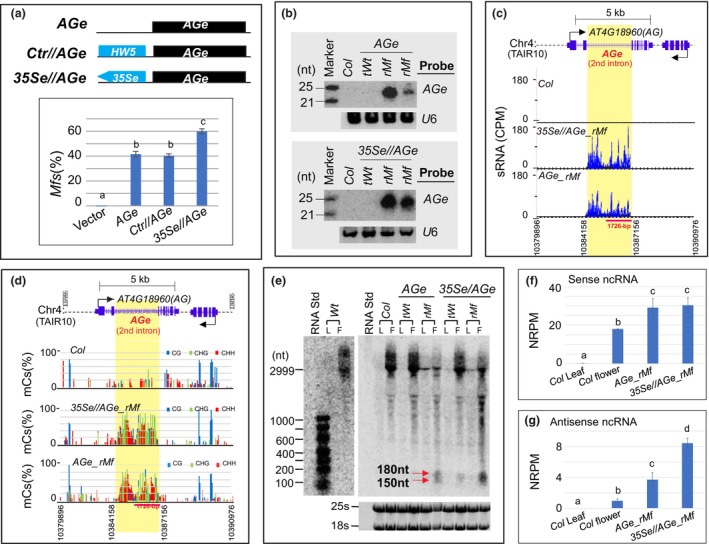
*35Se*‐mediated enhancement of mutant flower (*Mf*) production associated with siRNA production, DNA methylation and ncRNA transcription in the *AGe* region. (a) The effect of inserting the *35Se* immediately upstream of the *AGe* in the *35Se//AGe* cassette (top panel) on *Mf* production in transgenic lines (bottom panel). The frequency of *Mf* lines was averaged from three biological replicates (*n* > 50), with ±SD. Significant differences are indicated by asterisks among transgenic lines harbouring the empty vector, *AGe*, *Ctr//AGe* and *35Se//AGe* transgenes, respectively (*P* < 0.05, ANOVA). *HW5* – a control DNA fragment cloned from the *Arabidopsis* genome (see Experimental Procedures for detailed information). (b) sRNA gel blot for siRNA production in *rMfs*. (c, d) siRNA production (c) and cytosine methylation (d) along the *AGe* region in *rMfs*. Top panel – Gene annotation of the *AG* locus and *AGe* on *Arabidopsis* chromosome 4. (e) RNA gel blot for the detection of enhanced ncRNAs by the *35Se* in the *AGe* (See Figure 2 legend for detailed information). (f, g) Strand‐specific RNA‐seq analysis of sense (f) and anti‐sense (g) ncRNAs transcribed from the *AGe* in *Wt* leaves and flowers, and *rMf*s, respectively. Data were averaged from three biological replicates (*n* = 5 for each replicate), with ±SD. Significant differences are indicated by asterisks among leaf tissue, *Col/Wt* flowers, *AGe rMf* flowers, and *35Se//AGe rMf* flowers (*P* < 0.05, ANOVA).

To examine whether *CRE–CRE* interactions increase/activate the ectopic transcription of ncRNAs and RdDM/silencing in interacting *CRE* partners, we placed the *35Se*, which is known for its promiscuous interaction with adjacent *CREs*, including the *AGe*, even in tissues where they are not typically expressed (Gudynaite‐Savitch *et al*., 2009; Singer *et al*., 2010; Weigel *et al*., 2000), next to the *AGe* to create a *35Se//AGe* cassette (Figure 3a). We introduced this cassette into the *Col Arabidopsis* ecotype, along with two control (*AGe*, *Ctr//AGe*) cassettes (Figure 3a), respectively. Transgenic lines containing the *AGe* alone, or a 2‐kb control fragment inserted immediately upstream of the *AGe* (*Ctr//AGe*), produced *Mfs* in approximately 41% and 39% of lines, respectively. In contrast, the insertion of the *35Se* fragment next to the *AGe* (*35Se//AGe*) significantly increased this frequency to approximately 60% (*P* < 0.05, *t*‐test; Figure 3a). *rMfs* from those lines bearing either the transgenic *AGe* or *35Se//AGe* cassettes produced an abundance of 24‐nt siRNAs (Figure 3b,c) and displayed similar methylation patterns along the *AGe* region (Figure 3d). Much stronger ncRNA bands, including 150‐ and 180‐nt ncRNA species, were also observed in *35Se//AGe rMfs* compared to those produced in *AGe* lines, and this enhancement occurred only in the flowers (Figure 3e). This was further validated by ssRNA‐seq analysis, whereby *35Se//AGe rMfs* produced significantly more anti‐sense ncRNAs than *AGe* lines (*P* < 0.05; Figure 3g), while sense ncRNAs remained almost unchanged (Figure 3f), indicating that the *35Se*‐mediated ncRNA enhancement is flower‐specific and strand‐biased.

### *CaMV35S* promoter ( *35Sp* )‐transcribed RNAs from the *AGe* fail to enhance *Mf* production

To understand whether sense and anti‐sense RNAs transcribed by the *CaMV35S*/Pol II promoter (*35Sp*) also have a similar enhancing effect, we inserted the 3′ 1726‐bp *AGe* fragment downstream of the *35Sp* in sense and anti‐sense orientations to create *35Sp::sAGe* and *35Sp::asAGe* cassettes (Figure S4a), respectively. Surprisingly, neither *35Sp::sAGe* nor *35Sp::asAGe* transgenic populations showed an enhancement of *Mf* production, but instead displayed a drastic reduction (Figure S4a). This was particularly apparent in the *35Sp::asAGe* transgenic population, where only 5% of lines exhibited a *Mf* phenotype compared to approximately 41% of lines in the control transgenic population harbouring only the 3′ 1726‐bp *AGe* fragment (Figure S4a). The *rMfs* produced in both *35Sp::sAGe* and *35Sp::asAGe* lines, like those in *AGe* lines (Figure 1i), exhibited abundant levels of 24‐nt siRNAs, but no accumulation of larger RNAs (Figure S4b,c). Conversely, *tWt* flowers accumulated an abundance of large RNAs in both leaf and flower tissues, but no siRNAs compared to *Wt* flowers (*Col*, *Ler* and *WS*) and *rMfs* (Figure S4b,c). These results indicate that *35Sp*‐transcribed Pol II transcripts from the *AGe*, unlike *35Se*‐enhanced/activated ncRNAs, fail to feed and fuel siRNA biogenesis/RdDM activity.

### A *35Se* inserted into the *Arabidopsis* genome distally activates siRNA production and *de novo* methylation specifically in the *AGe* region, as well as *Mf* production

**Figure 4:**
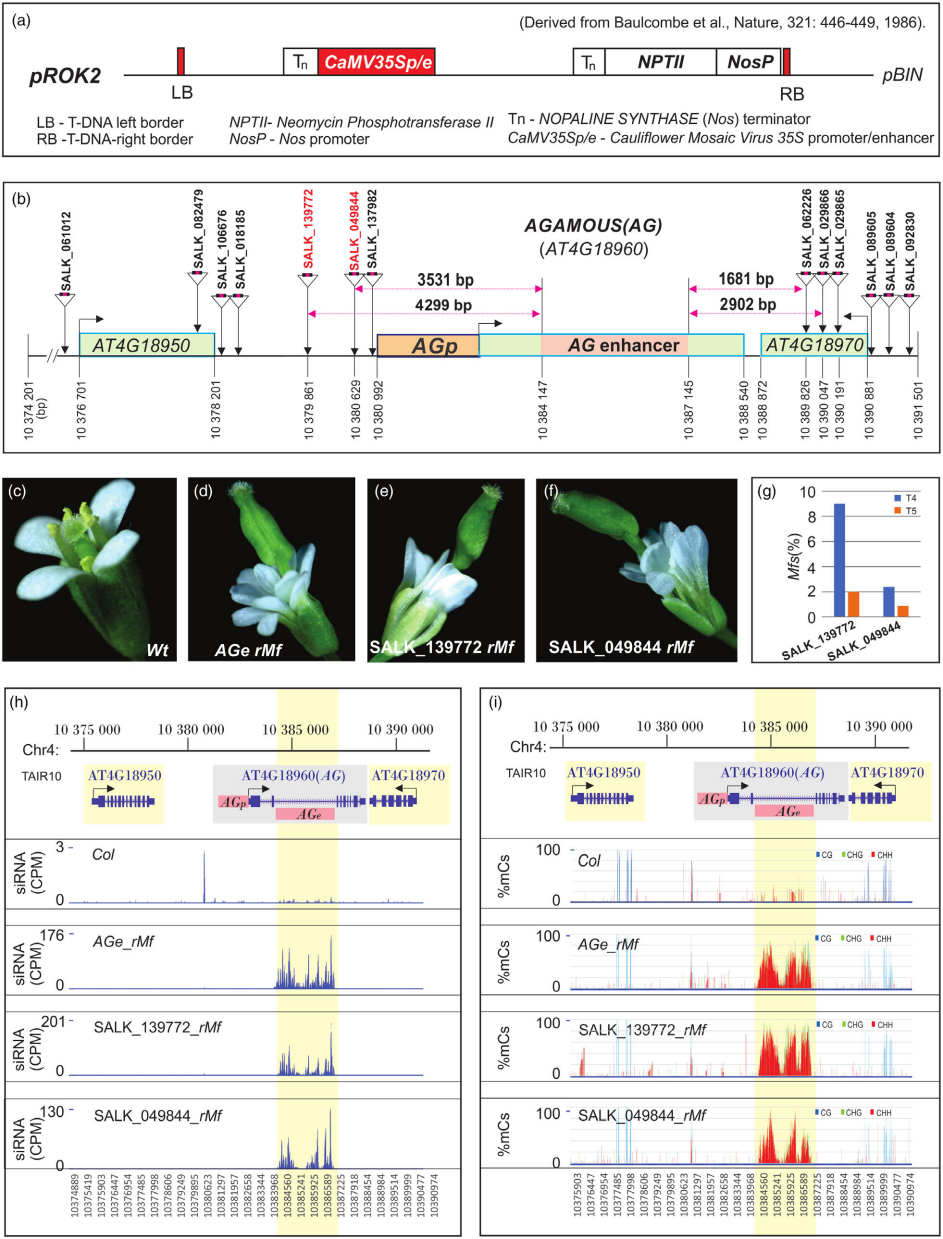
Activation of *Mf* production and RdDM activity in *Arabidopsis* lines bearing a *35Se* inserted at least 3.5 kb upstream of the native *AGe* in the genome. (a) Schematic illustration of the *pROK2* construct used for SALK T‐DNA insertion lines (Baulcombe *et al*., 1986). (b) Illustration of the genomic (TAIR10) positions of 13 T‐DNAs inserted up‐ and downstream of the *AG*‐transcribed region. Two lines, SALK_139772 and SALK_049844, which carry T‐DNAs inserted approximately 4.3‐kb and 3.5‐kb upstream of the *AGe*, respectively, and exhibit *Mf* production, are marked in red. Information regarding T‐DNA insertion positions and flanking sequences within the genome was obtained from the Salk Institute Genomic Analysis Laboratory accessible through the SIGnAL website http://signal.salk.edu. (c–f) *Wt* flowers (c) and *rMfs* from *AGe* transgenic (d), SALK_139772 (e) and SALK_049844 (f) lines. (g) *Mf* frequency in T_4_ and T_5_ generations of SALK_139772 and SALK_049844 lines. (h, i) *AGe*‐specific siRNA production (h) and methylation (i) in T_4_ *rMf*s of the indicated lines. Top panel – Gene annotation in the 17.3‐kb genome region containing *AG and* other loci, with *AGp* and *AGe* highlighted. CPM – Read count per million mapped reads.

We subsequently examined if the insertion of a *35Se* near the *AGAMOUS* (*AG*) locus in the *Arabidopsis* genome would lead to the activation of ncRNA/siRNA biogenesis and RdDM within its *AGe* and promoter regions, which are situated approximately 1.2 kb apart. Given that the *pROK2* binary vector, which harbours a *35Se* near its left border (Figure 4a; Baulcombe *et al*., 1986), was used for the generation of SALK T‐DNA lines (Alonso *et al*., 2003), we searched for the insertion of T‐DNA transgenes upstream and downstream of the *AG* locus in the SIGnAL database (http://signal.salk.edu) and identified at least 13 such T‐DNA insertion events (Figure 4b). We hypothesised that if the inserted *35Se* interacts with the *AGe* and activates ncRNAs/RdDM activity, some or all of these lines would produce flowers with similar *wMf* and *rMf* phenotypes. Of the 13 T‐DNA lines evaluated in the T_4_ generation, SALK_139772 and SALK_049844 lines exhibited *Mfs* (Figure 4e,f) similar to those induced by the *AGe* transgene (Figure 4d) in approximately 9% and 2.4% of each population, respectively (Figure 4g). Both of these lines carried T‐DNA insertions upstream of the *AG* promoter, approximately 4.3‐kb and 3.5‐kb from the *AGe*, respectively (Figure 4b). In the T_5_ generation, the formation of *Mfs* was reduced to less than 2% and 1% of the evaluated populations (Figure 4g), respectively. This inheritance pattern differs from typical genetic traits. The *Mf* lines analysed harboured the *35Se* fragment, as confirmed by PCR analysis (Figure S5), and produced abundant 24‐nt siRNAs and extensive methylation exclusively in the *AGe* region, but not in other regions surrounding the *AG* locus (Figure 4h,i). These findings indicate that the *35Se* specifically activated ncRNA/siRNA production and RdDM within the *AGe*.

## Discussion

Despite the well‐documented phenomenon of transgene silencing in plants, it is unclear why *CREs* become particularly susceptible to RdDM/silencing when in a transgenic state. We provide evidence that the transgene state‐dependent silencing of the *Arabidopsis AGe* is caused by the ectopic transcriptional activation of ncRNAs (Figures 2a and 3e–g), which is otherwise repressed in the *AGe*'s endogenous counterpart. Our findings further revealed that *AGe* transgene‐self‐transcribed ncRNAs, but not those ectopically transcribed by a Pol II *35Sp*, are potent enough to induce RdDM (Figure S4a–c), which is consistent with the fact that canonical RdDM is only activated by Pol IV‐derived, but not Pol II‐derived, RNA transcripts (Law and Jacobsen, 2010). The fact that the size of prominent 150‐ and 180‐nt ncRNA species produced in mutant flowers corresponds to the 90‐ to 150‐nt length in regions covering a series of discrete siRNA peaks generated along the *AGe* region (Figure 2i) suggests that these two ncRNA species serve as the primary source of 24‐nt siRNA precursors. Although Pol IV has previously been shown to transcribe shorter transcripts 30–45‐nt in length, which each encode a single siRNA (Blevins *et al*., 2015; Zhai *et al*., 2015), it also transcribes longer ncRNAs from both strands of tens of thousands of RdDM loci in the *Arabidopsis* genome, with the majority being 100‐ to 500‐nt in length (Li *et al*., 2015). Given that RdDM in the *AGe* requires Pol IV (Figure S3a), this polymerase is likely responsible for the ectopic transcription of the 150‐ and 180‐nt ncRNAs, as well as other ncRNAs within the *AGe*.

In the present study, our findings support the hypothesis that *CRE*–*CRE* interactions play a key role in inducing ncRNA transcription and RdDM activity in the endogenous and transgenic *AGe*. This was evidenced by the fact that placing a *35Se* next to the *AGe* in a transgene cassette significantly increased ncRNA/siRNA abundance and the frequency of the resulting mutant flower lines (Figure 3a–g). Furthermore, inserting the *35Se* approximately 3.5 kb upstream of the native *AGe* in the *Arabidopsis* genome also led to the *de novo* activation of siRNA production and DNA methylation, as well as similar mutant flower phenotypes (Figure 4b,e–i). Notably, this activation only occurred in the *AGe* and not in other regions of the *AG* locus, including the *AG* promoter (*AGp*) (Figure 4h,i), indicating that this *35Se*‐mediated activation of ncRNA transcription and ensuing RdDM is *AGe*‐specific. The *35Se* has been shown to actively interact with a variety of *CREs* or enhancers/promoters, including the *AGe*, resulting in the ectopic activation of the interacting partners' regulatory activities in tissues where they are not normally active (Gudynaite‐Savitch *et al*., 2009; Singer *et al*., 2010; Weigel *et al*., 2000; Zheng *et al*., 2007). Notably, our findings provide the first evidence that this *CRE‐CRE* or enhancer–enhancer interaction can also ectopically trigger ncRNA transcription, leading to RdDM and silencing in one of the associated *CREs* (Figure 4a).

This is a novel epigenetic feature not yet reported in plants and animals. However, it remains unclear why only two of thirteen SALK lines failed to produce *Mfs* in T_4_ plants. This was the case even though some of the lines (i.e. SALK_137982, SALK_062226, SALK_029866) carried T‐DNAs inserted closer to the native *AGe* than SALK_139772 and SALK_049844. Given that only T_4_ seeds were available and used for analyses, it is possible that these lines might have produced *Mfs* in T_1_ and T_2_ generations, but gradually lost this phenotype in subsequent generations. This scenario aligns with the observation that *Mf* frequency was drastically reduced from T_4_ to T_5_ generations in SALK_139772 and SALK_049844 lines (Figure 4g). Alternatively, it is also feasible that interaction between the *AGe* and *35Se* in these lines could be attenuated by unknown intervening genomic configurations or chromatin modifications, or specific sequence motifs that are bound by insulator factors or other protein factors.

Enhancers and promoters are known to interact to control the spatiotemporal and quantitative expression dynamics of their target genes (He *et al*., 2014; Kyrchanova *et al*., 2022; Ricci *et al*., 2019; Sanyal *et al*., 2012; Zhao *et al*., 2019, 2020). However, many plant *CREs* in enhancers or promoters frequently become, in the absence of a native genomic context, promiscuously interactive with adjacent *CREs*, leading to their non‐specific activation (Gudynaite‐Savitch *et al*., 2009; Wen *et al*., 2014). This is particularly evident in the case of transposon‐ and transgene‐derived *CREs*, which can interact with nearby *CREs* within the genome (Gudynaite‐Savitch *et al*., 2009; Hayashi and Yoshida, 2009; Hirsch and Springer, 2017; Hollister and Gaut, 2009; Kashkush, 2007; Kashkush *et al*., 2003; Matzke and Matzke, 1998; Wen *et al*., 2014). These promiscuous interactions are not limited to specific types of *CREs*, but can occur between constitutively active *CREs*, tissue‐specific *CREs* or a combination of both (Gudynaite‐Savitch *et al*., 2009; Liu *et al*., 2008; Singer *et al*., 2010; Wen *et al*., 2014). It is evident that transgenic *CREs* inserted into genomes inevitably interact with and activate, or are activated by, adjacent *CREs* in the genome. Such promiscuous interactions likely trigger ectopic transcription of ncRNAs, as seen in the *35Se‐AGe* interaction, making transgenic *CREs* susceptible to RdDM. However, not all *CREs* have this interactive capacity. For instance, while the seed‐specific soybean *Prx* promoter introduced into *Arabidopsis* within a transgene was previously shown to be ectopically activated by adjacent *CREs* in the genome, the seed‐specific *Brassica napus napin* promoter (*Pnapin*) was not affected by neighbouring *CREs* (Gudynaite‐Savitch *et al*., 2009). This difference in inherent *CRE* interactivity could explain the divergence in silencing vulnerability between resistant (*AP1p*, *LFYp* and *AGL8p*) and susceptible (*AGe*, *AP3p* and *SUPp*) transgenes, as well as the variation in silencing frequencies observed among *AGe* (42%), *AP3p* (44%) and *SUPp* (28%) transgenes (Table 1).

As of yet, it remains unclear exactly how the interaction between *CREs* activates the ectopic transcription of RdDM‐potent ncRNAs. In mammalian cells, ncRNAs are transcribed from *CREs* in enhancers and promoters in a coactivator‐ and transcription factor (TF)‐dependent manner to perform a diverse set of biological and molecular regulatory functions (Kristjansdottir *et al*., 2020; Lai *et al*., 2015; Wang *et al*., 2022). Interestingly, the floral organ‐specific expression conferred by the *AGe* is dependent on the binding of flower‐regulated transcription factors (TFs) including AP1, AP2, AP3 and LFY (Figures S1i and S2; Busch *et al*., 1999; Lohmann *et al*., 2001). In the presence of an adjacent *35Se*, *AGe* regulatory activity is ectopically activated in leaf, stem, root and other vegetative tissues (Singer *et al*., 2010), hinting at the possibility that TFs binding to the *35Se* interact with those binding to the *AGe* to activate the *AGe's* regulatory activity in vegetative tissues.

Interestingly, *35Se*‐mediated activation/enhancement of ncRNA transcription occurred in flowers but not in leaves (Figure 3e), suggesting that *AGe*‐bound transcription factors (TFs) may be involved in the flower‐specific ectopic activation of ncRNA transcription. Upon interaction with *35Se*‐bound TFs, *AGe*‐bound TFs may change their mode of regulatory action and directly engage with Pol IV instead of Pol II in the promoter, initiating the ncRNA transcription process within the *AGe*. Alternatively, these TF‐TF interactions could lead to a conformational change that allows *AGe* chromatin to be accessible to Pol IV, thus priming the RdDM pathway. It is also possible that such a conformational change could be recognised by a genome surveillance system as a compromised regulatory signal. This could lead to the recruitment of Pol IV and RdDM components. Furthermore, we cannot exclude the possibility that these TF‐TF interactions could override anti‐RdDM settings in native genomic contexts or in TF complexes in the *AGe*. Further research will be required to unravel the precise mechanism(s) responsible for this phenomenon.

The discovery of the activation of ncRNA transcription and RdDM activity by *35Se*‐*AGe* interactions sheds mechanistic light on the mystery of hypermethylation preferentially occurring in tandemly oriented transgenes, duplicate genes/enhancers and repetitive genomes rich in transposable elements. All of these configurations share a common feature: *CRE* duplication. This type of duplication has been demonstrated to lead to strong interactions between *CREs*, resulting in robust increases in the expression of target genes (Belele *et al*., 2013; Kay *et al*., 1987; Loehlin *et al*., 2022; Loehlin and Carroll, 2016). Such interactions between duplicate enhancers are also essential for the induction of hypermethylation during paramutation in loci such as maize *b1*. In this particular instance, an 843‐bp enhancer located approximately 100‐kb away regulates the *b1* gene, which confers red pigment production in an enhancer copy number‐dependent manner as tandemly duplicated enhancers induce strong dark red pigment production while a single copy does not (Arteaga‐Vazquez and Chandler, 2010; Hollick, 2017; Stam *et al*., 2002). However, maize plants with tandemly duplicated enhancers, but not those with a single copy of the enhancer, also experience frequent silencing or paramutation of *b1* (Arteaga‐Vazquez *et al*., 2010). This enhancer duplication‐dependent paramutation relies on the ectopic activation of ncRNA transcription, siRNA production and methylation in tandemly duplicated enhancers, and requires components of the canonical RdDM pathway (Alleman *et al*., 2006; Arteaga‐Vazquez *et al*., 2010; Belele *et al*., 2013; Haring *et al*., 2010; Stam *et al*., 2002). Thus, this duplicate enhancer configuration‐dependent induction of RdDM/paramutation in the *b1* locus appears to share the same causal force as the *35Se‐AGe* interaction‐mediated activation of RdDM in the *AGe*. This suggests that ectopic *CRE–CRE* interactions may also drive the methylation vulnerability of tandemly duplicated genes and repetitive transposon‐rich regions in plant genomes.

In conclusion, our findings demonstrate that *35Se‐AGe* interactions lead to ncRNA production and RdDM activity, highlighting a novel epigenetic role for ectopic *CRE‐CRE* or enhancer–enhancer interactions in plants. This provides fresh insight into the driving forces behind methylation susceptibility in transgenes – a phenomenon that may also explain hypermethylation propensity in duplicated enhancers/genes and repetitive transposons, where *CRE–CRE* interactions are inevitable.

## Experimental procedures

### Plasmid constructs, plant transformation and tissue collection

A 2270‐bp *AGL8* promoter (*AGL8p*), 1746‐bp *LFY* promoter (*LFYp*), 1080‐bp *AP3* promoter (*AP3p*), 6600‐bp *SUPERMAN* promoter (*SUPp*) and 2999‐bp *AG* enhancer (*AGe*) fragment were amplified from the *Arabidopsis* Col ecotype using the primers listed in Table S1. The resulting amplicons were end‐filled using DNA polymerase/Klenow fragment (NEB, Ipswich, MA) and inserted into the *Sma* I site in the *pBIN19* vector to create *pAGL8p*, *pLFYp*, *pAP3p*, *pSUPp* and *pAGe*, respectively (Figure S1a). In contrast, the pAP1p vectors, which were originally acquired from Dr. Martin Yanofsky, were created by inserting a 3360‐bp fragment encompassing the 1605‐bp *AP1* promoter (*AP1p*) along with the first exon, first intron and second exon into the *Hind* III and *Xba* I sites of the *pDW137* binary vector (see diagrammatic illustration in Figure S1a). The *AGe* deletion derivatives in vectors *pR2509*, *JM43*, *pR2088* and *JM44* were generated through restriction digestion and re‐ligation (Figure S1i). The *pR2509* vector includes a 2181‐bp 5′ region of the *AGe*, the *JM44* vector contains an 818‐bp 3′ fragment of the *AGe*, the *pR2088* vector contains a 908‐bp centrally‐located region of the *AGe*, and the *JM43* vector includes a 1726‐bp 3′ region of the *AGe* (Figure S1i). An 80‐bp fragment was removed from *JM43* to create *JM94* to allow for PCR‐based differentiation between native and transgenic *AGe*‐derived transcripts.

The 1726‐bp 3′ *AGe* fragment from *JM43* was inserted between the *CaMV35S* promoter and *Nos* terminator in the *pR375* binary plasmid to create *35Sp::sAGe* and *35Sp::asAGe* vectors, respectively. *35Se//AGe* and *Ctr//AGe* constructs were created by inserting a 510‐bp fragment containing a tandem duplication of the 253‐bp *CaMV35S* enhancer (Kay *et al*., 1987) between the −343 and −90 positions in the *CaMV35S* promoter and a 2029‐bp control fragment (*HW5*) upstream of the *AGe* in the *pAGe* vector (Figure 3a), respectively. The *HW5* fragment was cloned from *Arabidopsis* chromosome 3 between genome coordinates 20 126 189 and 20 124 221 (GenBank accession cp002686.1). Further details regarding cloning are available upon request.

*Arabidopsis* transformation was performed using the floral dip method (Clough and Bent, 1998). Transgenic T_1_ plants were grown under a 16/8 h light:dark photoperiod at 20–22 °C for 4–5 weeks before evaluation of flower phenotypes at 23–25 °C from weeks 6 to 8. All SALK_T‐DNA lines (T_4_ generation) were ordered from the *Arabidopsis* Biological Resource Center/ABRC (https://abrc.osu.edu) and information regarding T‐DNA insertion sites was obtained from the public T‐DNA Express database accessible on the SIGnAL website (http://signal.salk.edu).

Young, open flowers were collected from 5 to 20 individual *Wt* or T_1_ transgenic lines that displayed true flower phenotypes over a growth period. They were pooled together in equal amounts for RNA gel blots, semi‐quantitative strand‐specific RT‐PCR and genome‐wide methylation analyses. For statistical analysis, three replicates of flower tissue samples, each containing tissues pooled from at least five lines, were prepared.

#### sRNA gel blot

Total RNA was extracted from the collected flower tissues using Tri® Reagent (Sigma‐Aldrich, Sigma, MO) as described previously (Xia *et al*., 2012). Approximately 30 μg of total RNA were separated through a 16% denaturing polyacrylamide gel, along with miRNA markers (0.1, 0.25, 0.5, and 1 ng, see Figure S1c,e,g) and electroblotted onto a Hybond‐NX membrane (Amersham, NJ) before co‐hybridisation with a α‐^32^P dCTP‐labelled gene‐specific probe and γ‐^32^P ATP‐labelled miRNA marker (approximately 1/20 labelled reaction was added to the hybridisation solution). To re‐probe, the same filter was stripped by boiling in 1% SDS and 5 mM EDTA for 10–30 min and exposed for at least 24 h to ensure that the signal from the previously hybridised radioactive probe was completely removed before co‐hybridisation with γ‐^32^P ATP‐labelled *U6*, miR319 and miRNA marker in a ratio of 1 : 1 : 1/50 labelled reactions. The random primer labelling kit (Invitrogen, CA) was used for ^32^P‐labelled of the *AGe*, *AP3p* and *SUPp* fragments, respectively. The 3′ end labelling of oligonucleotide probes *U6*, *IR‐71, AtSN1*, miR319 and the miRNA marker (NEB, MA) was carried out using T4 polynucleotide kinase (NEB, MA) in the presence of 0.5 μCi/μL of (γ‐^32^P)ATP. Table S1 lists all oligonucleotide sequences.

#### Regular RNA gel blot

Approximately 30 μg of total RNA were separated on a 1.5% agarose gel. An RNA marker ranging in length from 100‐nt to 1000‐nt (Novagen, WI) was also included. The gel was blotted onto a nylon filter, followed by UV crosslinking, and hybridised according to the NorthernMax‐Gly kit (Ambion, TX). The filter was first hybridised with the ^32^P‐labelled 3′ 1726‐kb *AGe* probe, and washed in 2X SSC, 0.1% SDS at 50 °C for 30 min once, and 0.1X SSC, 0.1% SDS at 55 °C for 60 min twice. The same filter was then re‐probed with a ^32^P‐labelled RNA marker template (2 μL of 50 μL labelling mix) without stripping. The random primer labelling kit (Invitrogen, CA) was used for ^32^P‐labelling of both the *AGe* fragment and the RNA marker template (Novagen, WI).

#### *In situ* hybridisation

Clusters of unopened flower buds from *Col* wild‐type and *AGe*‐transformed lines with *Wt* and strong mutant flower phenotypes were collected from 4 to 5 week‐old plants, fixed and embedded as described previously (Drews *et al*., 1991). Tissue blocks were cut into 8‐mm longitudinal sections using a microtome (LKB Ultratome V, LKB Bromma, Sweden) and attached to Superfrost Plus slides (Fisher Scientific, Hampton, NH). *AG*‐specific anti‐sense RNA and sense RNA probes were prepared by amplifying a 489‐bp fragment of *AG* cDNA using primers AGprobeF1 and AGprobeT7R1 and AGprobeT7F1 and AGprobeR1 (Table S1). PCR fragments were purified and labelled using *in vitro* transcription reactions with DIG‐labelled NTPs along with T7 RNA polymerase according to the manufacturer's recommendations (Roche). Transcripts were purified using the RNeasy MinElute Cleanup Kit (Qiagen). *In situ* hybridisations were performed as described previously (Karlgren *et al*., 2009).

#### Semi‐quantitative strand‐specific RT‐PCR

Total RNA was isolated from *Wt* plants and *JM94* T_1_ transgenic lines, and was assessed via PCR to ensure that all DNA had been removed following DNase treatment. Subsequently, the *AGe* sense and anti‐sense transcripts were converted to cDNA using Superscript III (Invitrogen) with primers P2/AGI‐IIR7 (specific for sense transcripts), P1/AGI‐IIF6 (for anti‐sense transcripts) or poly(dT) primer (for the EF1α internal RNA control), respectively (primer sequences are listed in Table S1). Subsequent amplifications were carried out using GoTaq HotStart polymerase (Promega) in a final volume of 50 μL, at 95 °C for 2 min followed by 24–35 cycles of 95 °C for 30 s, 59 °C for 30 s and 72 °C for 30 s. The P1/AGI‐IIF6 and P2/AGI‐IIR7 primer pairs are expected to amplify a 380‐bp fragment from endogenous *AGe* transcripts and a 300‐bp fragment from *JM94*‐containing *AGe* transgenic transcripts (see illustration in Figure 2b).

#### PCR bisulphite sequencing

Analysis of DNA methylation within an endogenous and transgenic 1726‐bp *AGe* region was performed as described previously (Henderson *et al*., 2010). Pure, high‐quality genomic DNA from *Col Wt* and transgenic (*JM43*) mutant flowers was isolated using the Qiagen DNeasy Kit (Qiagen, MD). Approximately 300 ng of genomic DNA was converted using the EZ DNA methylation kit (Zymo Research, CA). Two primer pairs (EF1 and R4, and F4 and ER2; Table S1) were designed and employed for the amplification of the 1726‐bp endogenous *AGe* fragment, while primer pairs F1 and R4, and F4 and R7 (Table S1) were used for amplification of the transgenic *AGe*. PCR amplification was carried out at 95 °C for 30s, 53 °C for 90s, and 72 °C for 3 min with 35 cycles. The amplified fragments were cloned into the pGEMT‐Easy vector (Promega, WI), and 20 colonies from each ligation were chosen for sequencing. The frequency and pattern of CG, CHG and CHH methylation were calculated at each position.

#### Whole genome bisulphite sequencing

Genomic DNA isolated from collected flowers was submitted to BGI Americas Cooperation (Cambridge, MA) for library construction and whole genome bisulphite sequencing, with at least 30X genome coverage data obtained for each sample. The error conversion rates were below 0.005% for unmodified cytosines, and sequencing quality was evaluated using FastQC (version 0.11.9) (https://www.bioinformatics.babraham.ac.uk/projects/fastqc/). FASTQ files were aligned to the TAIR10/Araport11 *Arabidopsis* reference genome (Cheng *et al*., 2017) using the Bismark Bisulfite Read Mapper with the Hisat2 alignment algorithm (Krueger and Andrews, 2011). The methylation status of each cytosine in each context was extracted from the alignments with the biomark methylation extractor. The percent of methylated cytosines (%mCs) in each position where the total read depth was at least 4 reads, out of the total number of cytosines, was calculated in 17.3‐kb regions containing the *AG* locus and exported into Excel for graph preparation.

#### Small RNA‐seq analysis

One microgram of total RNA was used for the generation of small RNA libraries using the NEXTflex™ Small RNA‐Seq Kit v3 (BIOO Scientific, Austin, Texas) according to the manufacturer's instructions. The resulting libraries were sequenced using a HTIseq4000 by BGI Americas (Boston, MA). Clean reads were aligned to the reference *Arabidopsis* genome (TAIR10) using Bowtie software (version 2.4.1). Aligned reads in the *AG* region were counted using htseq‐count (version 0.13.5), with the bin size set to 1 to normalise the number of reads per bin for the calculation of counts per million mapped reads (CPM).

#### Strand‐specific RNA‐seq analysis

One microgram of total RNA was treated with Turbo DNA‐free (Fisher Scientific) to remove residual genomic DNA. Ribosomal RNA depletion, first‐ and second‐strand cDNA synthesis, 3′ end adenylation, adapter ligation, and DNA fragment enrichment (12 cycles) were carried out following the TruSeq stranded total RNA protocol (Illumina). Final libraries were examined on an agarose gel, quantified, pooled and sequenced by BGI Americas (Cambridge, MA). The filtered reads were mapped to the reference genome (TAIR10) using HISAT2 with default parameters (Kim *et al*., 2015), and mapped reads in the *AGe* region were extracted and counted using htseq‐count (version 0.13.5) (Anders *et al*., 2015). BamCoverage in Deeptools software (version 3.5.1) (Ramirez *et al*., 2014) was used to convert aligned reads in BAM format to BigWig format for visualisation in the local UCSC genome browser (Kent *et al*., 2002), with the bin size set to 1 to normalise the number of reads per bin for the calculation of counts per million mapped reads (CPM). The values of normalised reads per million mapped reads (NRPM) were calculated specifically for sense and anti‐sense transcripts produced in the *AGe* region.

#### Verification of transgenes in SALK_T‐DNA lines

Approximately 200 ng of DNA was used for the amplification of *35Se*, *NPTII* and *EF1* fragments using 35SF1 and 35SF2, NPTIIF1 and NPTIIR2, and EF1F and EF1R primer pairs (Table S1), respectively. Thermal parameters for PCRs were 95 °C for 2 min, 28 cycles of 95 °C for 1 min, 58–62 °C for 30s and 72 °C for 1 min, followed by a final extension at 72 °C for 10 min.

#### Statistical analysis

Data/NRPM values from three biological replicates were averaged and presented as the mean ± standard deviation (SD). Significant differences (*P* < 0.05) between treatments were analysed using student's *t*‐tests while ANOVA was applied for significant differences (*P* < 0.05) among multiple treatments.

## Accession numbers

All sRNA‐seq, BS‐seq and ssRNA‐seq data have been deposited in NCBI's Gene Expression Omnibus and are accessible through GEO SuperSeries accession number GSE228431.

## Author contributions

YZhou Y, S.S., G.Y., D.R.B, J.M.H., W.X., X.W, Zchi L. and ZRang L carried out transgenic experiments and gene expression analyses. YZhou Y., J.L., YZhen Y., S.S., J.L., G.‐Y. Z, D.R.B. and ZRang L. conducted sRNA and ncRNA analyses. J.L., D.R.B. and ZRang L. performed WGBS‐seq. Y.L., Y.C.A. and ZRang L. carried out bioinformatic analysis. ZRang L. and S.S. wrote the manuscript. ZRang L. conceived and coordinated the research.

## Conflict of interest

The authors declare no competing interests.

## Supporting information


**Figure S1** Transgene silencing and mutant flower phenotypes.

**Figure S2** Transcription factors binding to the *AG* enhancer/*AGe* region on *Arabidopsis* chromosome 4 as revealed from meDIP and ChIP‐seq datasets (SRA accession numbers shown on the left).

**Figure S3** Characterisation of the RdDM pathway underlying *AGe* transgene silencing.

**Figure S4** *35S* promoter (*35Sp*)‐transcribed sense and anti‐sense RNAs failed to enhance *Mf* production.

**Figure S5** Association of the *35Se* with the *rMf* phenotype in two SALK_T‐DNA lines.

**Table S1** Sequences of primers utilised in this study.

## Data Availability

The data that support the findings of this study are openly available in NCBI's Gene Expression Omnibus or GEO at https://www.ncbi.nlm.nih.gov/geo/, reference number GSE228431.
