# Supplementary material for: Ectopic enhancer–enhancer interactions as causal forces driving RNA‐directed DNA methylation in gene regulatory regions
Source: Plant Biotechnol J. 2024 Jul 17;22(11):3121–34. doi: 10.1111/pbi.14435 (PMC11500991; doi:10.1111/pbi.14435)
Supplement: Supplementary file 6 — Table S1 Sequences of primers utilised in this study. [file PBI-22-3121-s003.docx]

**Table S1. Sequences of primers utilized in this study.**

| Primer name | Sequence (5’ – 3’) |
| --- | --- |
| P1/AGI-IIF6 | CTTTGGTGTAGATAGATTTATGC |
| P2/AGI-IIR7 | CGAGTAACATCACAACGTTCC |
| AGI-IIU80 | TCTAGAGAGAGAATAATGTTA |
| AGI-IIL80 | CCGTACTAAAAATCTCACTTTC |
| AGIntU693 | GAAATCTGGGAGAGGAAAGATCGA |
| AGIntL3962 | CATTAATTTCTGCCAGATATCCGGTGT |
| AGL5U | CTGTGACGTTGGATATATACACAAAAGCTTGAA |
| AGL5L1996 | TTTCTTTTAGAGGAAAGCTTCTTTGGAAGT |
| AGL8U | CCCAAATTTCCAACACAAGCTTCGAATTTAAATCTATTCTGAGT |
| AGL8L | TCTCTTCGAATTCTCAAGAGCTAGCGAGAGAAACGACAACCCT |
| AGPU5 | ACCTTAAATGGGTTCATAAAGCTTGAGAT |
| AGPL1580 | GATTGACATAGAGCAATCTAAAGGATCCCA |
| AGprobeF1 | CAATATCGGACAATTCTAACACC |
| AGprobeR1 | TAACTGGAGAGCGGTTTGGTC |
| AGprobeT7F1 | TAATACGACTCACTATAGGGCAATATCGGACAATTCTAACACC |
| AGprobeT7R1 | TAATACGACTCACTATAGGGTAACTGGAGAGCGGTTTGGTC |
| AP3U610B | TACCTTAGCCATAGGATCCCGTCTTGTAGATCT |
| AP3L1730Sal | AGATGGAGTTGAAGAAGTAAAGGGTCGACTT |
| AtSN1-LNA | ACCAACGTGTTGTTGGCCCAGTGGTAAATCTCTCAGATAGAGG |
| EF1 | TAATTGAATTTTTAAGATTTTAAATAG |
| ER2 | CTTACCTATACATTAATTTCTACCAC |
| EF1F | GCACTGTCATTGATG CTCC |
| EF1R | GTCAAGAGCCTCAAG GAGAG |
| F1 | ATAGGAAATAGTTATGATTATGATTA |
| F4 | TAGTTTTTGATTTTTGATAAGAAGGTT |
| IR-71-LNA | GGCTGCAACTCTTGCGGTAGAAGCCAATATGAGGAA |
| LFYU1710Bam | CAGACTGAGAGCATTAAGGATCCCAGTCTCT |
| LFYL1Sal | ATAATCTATTTTTCTCTCTCTCTCGTCGACTCTCT |
| PPVCPagU | AACACGGGTAGTCTAGACAGTCTTGTTTCCAAACTTGGTAT |
| PPVagL | GTGGGTTTCGCAGGATCCAACAATGGCT |
| R4 | ACTTCAAAAAAAAAAAAAAATTACAT |
| R7 | CCAAAATTTTCCCAATCA |
| SupU60B | TATTGAAGTTGATGTGGATCCAACAAATCT |
| SupL6630LSal  U6 | ATAACGTTCTACTAAATAGTCGACTTAGAGAAT  GCTAATCTTCTCTGTATCGTTCC |
| 35SF1  35SF2  NPTIIF1  NPTIIR2  HW5U  HW5D | ATGATGGCATTTGTAGGAG  ATTGTGCGTCATCCCTTAC  TCATCGCAAGACCGGCAACAG  AGGATGATCTGGACGAAGAGC  TCCATTTCAAGTAGTGCTAGACC  TGTAGAGTTCACCAACAGTGAGC |
