# Supplementary material for: Ectopic enhancer–enhancer interactions as causal forces driving RNA‐directed DNA methylation in gene regulatory regions
Source: Plant Biotechnol J. 2024 Jul 17;22(11):3121–34. doi: 10.1111/pbi.14435 (PMC11500991; doi:10.1111/pbi.14435)
Supplement: Supplementary file 2 [file PBI-22-3121-s001.docx]

# Fig S4

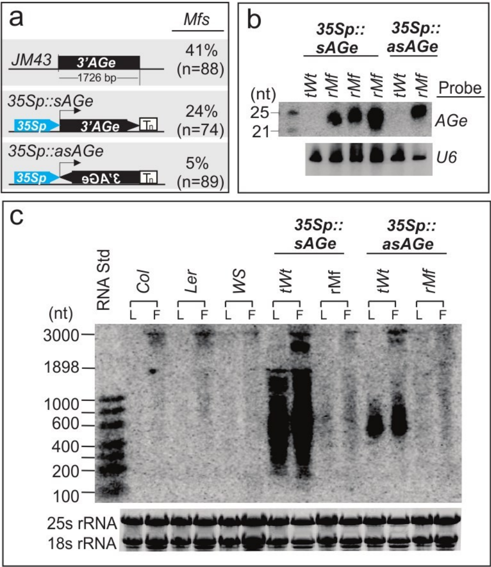

a
Mfs
JM43
3'AGe
1726 bp
41% (n=88)
35Sp::sAGe
35Sp
3'AGe
Tn
24% (n=74)
35Sp::asAGe
35Sp
3'AGe
Tn
5% (n=89)
b
35Sp:: sAGe
35Sp:: asAGe
tWt
rMf
rMf
rMf
tWt
rMf
Probe
(nt)
25
21
AGe
U6
c
35Sp:: sAGe
35Sp:: asAGe
RNA Std
Col
Ler
WS
tWt
rMf
tWt
rMf
L F
(nt)
3000
1898
1000
600
400
200
100
25s rRNA
18s rRNA
