# Supplementary material for: Ectopic enhancer–enhancer interactions as causal forces driving RNA‐directed DNA methylation in gene regulatory regions
Source: Plant Biotechnol J. 2024 Jul 17;22(11):3121–34. doi: 10.1111/pbi.14435 (PMC11500991; doi:10.1111/pbi.14435)
Supplement: Supplementary file 3 [file PBI-22-3121-s002.docx]

Fig. S2

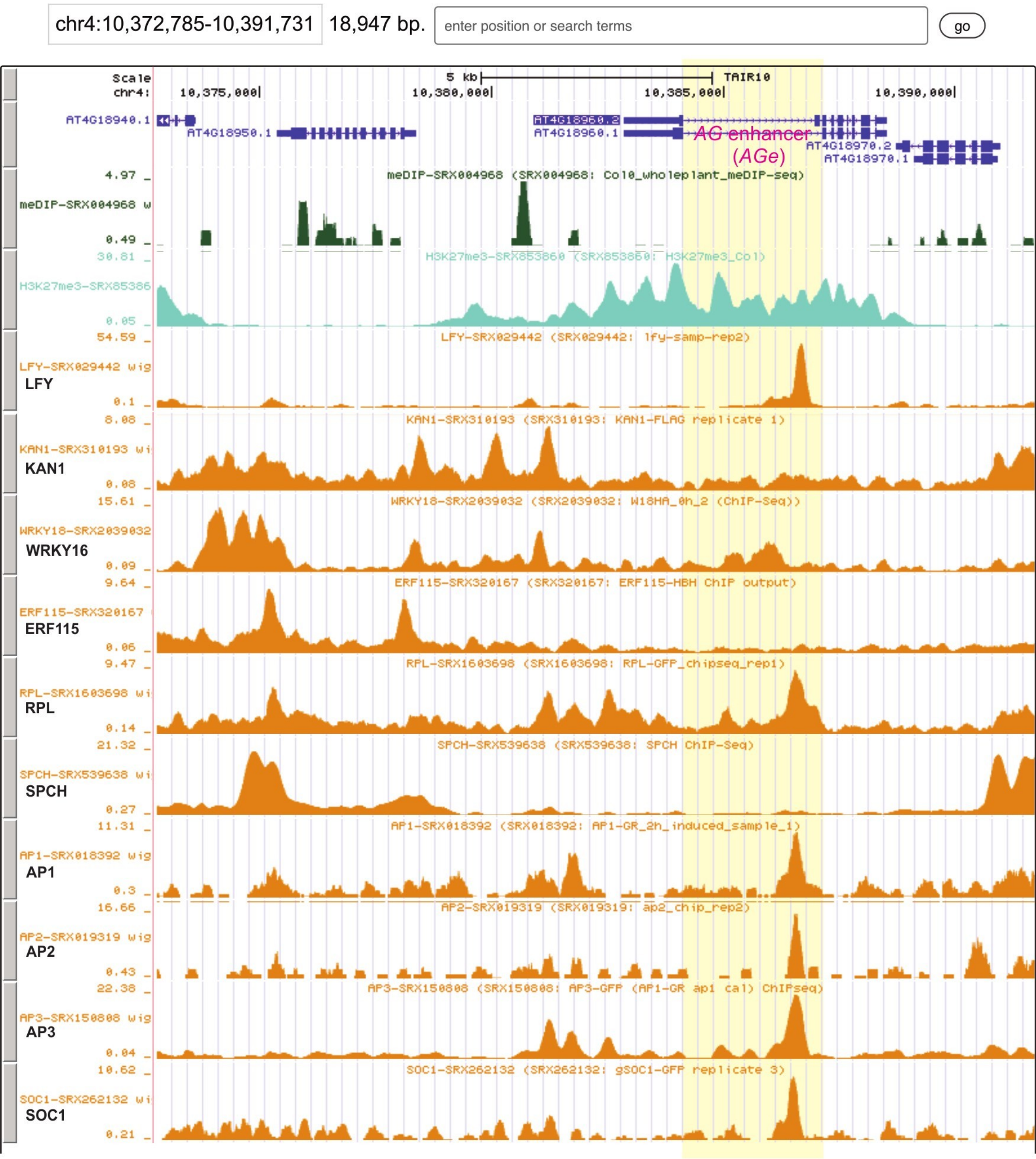

chr4:10,372,785-10,391,731
18,947 bp.
enter position or search terms
go
Scale
chr4:
5 kb
TAIR10
10,375,000
10,380,000
10,385,000
10,390,000
AT4G18940.1
AT4G18950.1
AT4G18960.2
AT4G18960.1
AG enhancer
(AGe)
AT4G18970.2
AT4G18970.1
meDIP-SRX004968 (SRX004968: Col0_wholeplant_meDIP-seq)
H3K27me3-SRX853860 (SRX853860: H3K27me3_Col)
LFY-SRX029442 (SRX029442: lfy-samp-rep2)
LFY
KAN1-SRX310193 (SRX310193: KAN1-FLAG replicate 1)
KAN1
WRKY18-SRX2039032 (SRX2039032: W18HA_0h_2 (ChIP-Seq))
WRKY16
ERF115-SRX320167 (SRX320167: ERF115-HBH ChIP output)
ERF115
RPL-SRX1603698 (SRX1603698: RPL-GFP_chipseq_rep1)
RPL
SPCH-SRX539638 (SRX539638: SPCH ChIP-Seq)
SPCH
AP1-SRX018392 (SRX018392: AP1-GR_2h_induced_sample_1)
AP1
AP2-SRX019319 (SRX019319: ap2_chip_rep2)
AP2
AP3-SRX150808 (SRX150808: AP3-GFP (AP1-GR ap1 cal) ChIPseq)
AP3
SOC1-SRX262132 (SRX262132: gSOC1-GFP replicate 3)
SOC1
