# Supplementary material for: Ectopic enhancer–enhancer interactions as causal forces driving RNA‐directed DNA methylation in gene regulatory regions
Source: Plant Biotechnol J. 2024 Jul 17;22(11):3121–34. doi: 10.1111/pbi.14435 (PMC11500991; doi:10.1111/pbi.14435)
Supplement: Supplementary file 4 [file PBI-22-3121-s004.docx]

Fig. S3

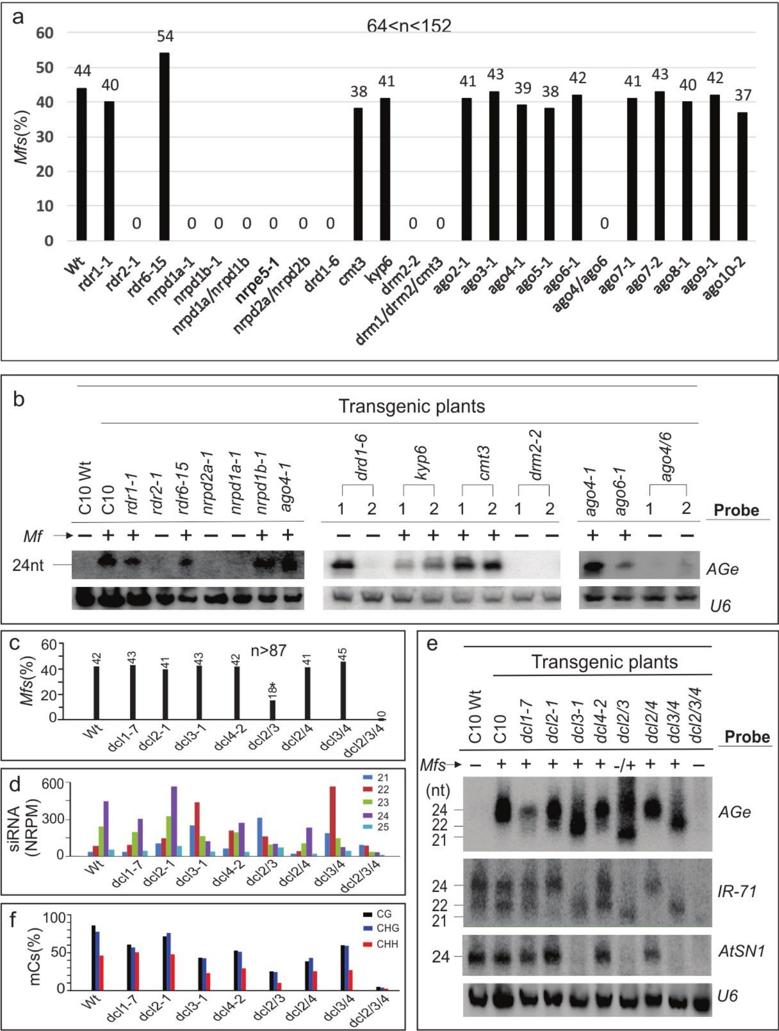
a
64<n<152
Mfs(%)
60
50
40
30
20
10
0
44
40
0
54
0
0
0
0
0
0
38
41
0
0
41
43
39
38
42
0
41
43
40
42
37
Wt
rdr1-1
rdr2-1
rdr6-15
nrpd1a-1
nrpd1b-1
nrpd1a/nrpd1b
nrpe5-1
nrpd2a/nrpd2b
drd1-6
cmt3
kyp6
drm2-2
drm1/drm2/cmt3
ago2-1
ago3-1
ago4-1
ago5-1
ago6-1
ago4/ago6
ago7-1
ago7-2
ago8-1
ago9-1
ago10-2
b
Transgenic plants
C10 Wt
C10
rdr1-1
rdr2-1
rdr6-15
nrpd2a-1
nrpd1a-1
nrpd1b-1
ago4-1
drd1-6
kyp6
cmt3
drm2-2
ago4-1
ago6-1
ago4/6
1 2 1 2 1 2 1 2 1 2
Probe
Mf
24nt
AGe
U6
c
n>87
Mfs(%)
60
40
20
0
42
43
41
43
42
18*
41
45
0
Wt
dcl1-7
dcl2-1
dcl3-1
dcl4-2
dcl2/3
dcl2/4
dcl3/4
dcl2/3/4
d
siRNA (NRPM)
600
300
0
21
22
23
24
25
Wt
dcl1-7
dcl2-1
dcl3-1
dcl4-2
dcl2/3
dcl2/4
dcl3/4
dcl2/3/4
f
mCs(%)
100
50
0
CG
CHG
CHH
Wt
dcl1-7
dcl2-1
dcl3-1
dcl4-2
dcl2/3
dcl2/4
dcl3/4
dcl2/3/4
e
Transgenic plants
C10 Wt
C10
dcl1-7
dcl2-1
dcl3-1
dcl4-2
dcl2/3
dcl2/4
dcl3/4
dcl2/3/4
Probe
Mfs
– + + + + + -/+ + + –
(nt)
24
22
21
AGe
24
22
21
IR-71
24
AtSN1
U6
