# Supplementary material for: Ectopic enhancer–enhancer interactions as causal forces driving RNA‐directed DNA methylation in gene regulatory regions
Source: Plant Biotechnol J. 2024 Jul 17;22(11):3121–34. doi: 10.1111/pbi.14435 (PMC11500991; doi:10.1111/pbi.14435)
Supplement: Supplementary file 5 [file PBI-22-3121-s005.docx]

Fig. S5

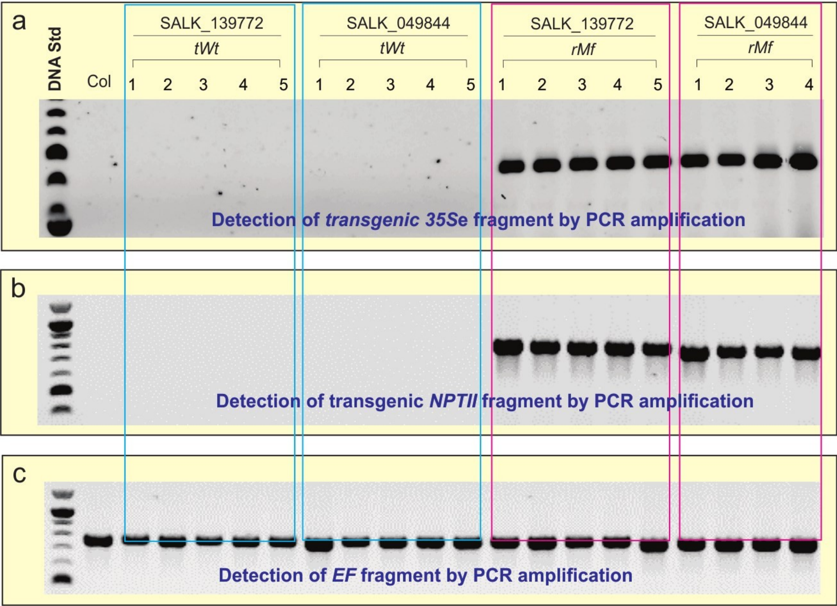
a
DNA Std
Col
SALK_139772
tWt
1 2 3 4 5
SALK_049844
tWt
1 2 3 4 5
SALK_139772
rMf
1 2 3 4 5
SALK_049844
rMf
1 2 3 4
Detection of transgenic 35Se fragment by PCR amplification
b
Detection of transgenic NPTII fragment by PCR amplification
c
Detection of EF fragment by PCR amplification
