# Supplementary material for: Ectopic enhancer–enhancer interactions as causal forces driving RNA‐directed DNA methylation in gene regulatory regions
Source: Plant Biotechnol J. 2024 Jul 17;22(11):3121–34. doi: 10.1111/pbi.14435 (PMC11500991; doi:10.1111/pbi.14435)
Supplement: Supplementary file 6 [file PBI-22-3121-s006.docx]

Fig. S1

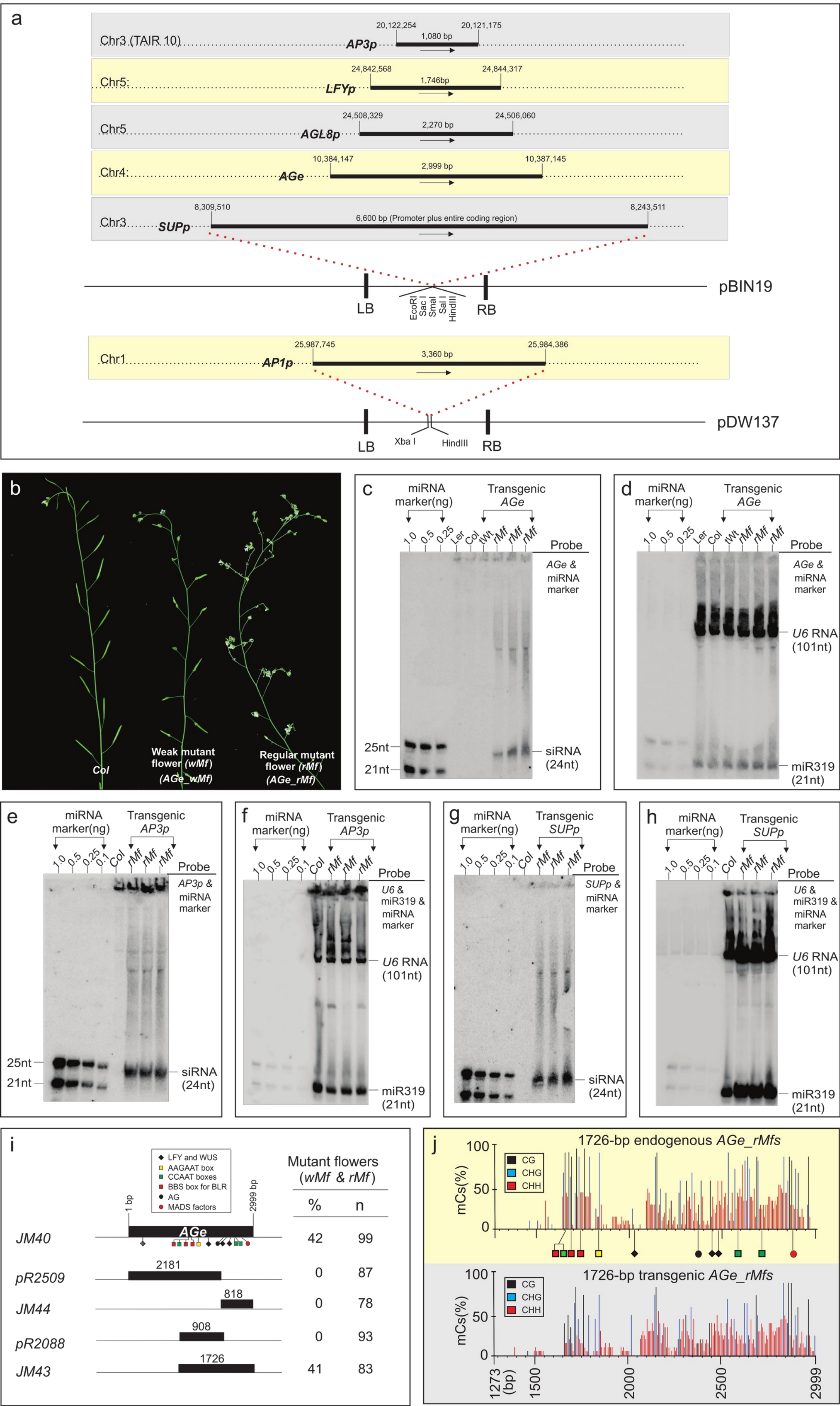

a
Chr3 (TAIR 10)
AP3p
20,122,254
20,121,175
1,080 bp
Chr5:
LFYp
24,842,568
24,844,317
1,746bp
Chr5
AGL8p
24,508,329
24,506,060
2,270 bp
Chr4:
AGe
10,384,147
10,387,145
2,999 bp
Chr3
SUPp
8,309,510
8,243,511
6,600 bp (Promoter plus entire coding region)
pBIN19
LB
RB
EcoRI
Sac I
SmaI
Sal I
HindIII
Chr1
AP1p
25,987,745
25,984,386
3,360 bp
pDW137
Xba I
HindIII
b
Col
Weak mutant flower (wMf) (AGe_wMf)
Regular mutant flower (rMf) (AGe_rMf)
c
miRNA marker(ng)
Transgenic AGe
1.0 0.5 0.25 Ler Col tWt rMf rMf rMf
Probe
AGe & miRNA marker
25nt
21nt
siRNA (24nt)
d
miRNA marker(ng)
Transgenic AGe
1.0 0.5 0.25 Ler Col tWt rMf rMf rMf
Probe
AGe & miRNA marker
U6 RNA (101nt)
miR319 (21nt)
e
miRNA marker(ng)
Transgenic AP3p
1.0 0.5 0.25 0.1 Col rMf rMf rMf
Probe
AP3p & miRNA marker
25nt
21nt
siRNA (24nt)
f
miRNA marker(ng)
Transgenic AP3p
1.0 0.5 0.25 0.1 Col rMf rMf rMf
Probe
U6 & miR319 & miRNA marker
U6 RNA (101nt)
miR319 (21nt)
g
miRNA marker(ng)
Transgenic SUPp
1.0 0.5 0.25 0.1 Col rMf rMf rMf
Probe
SUPp & miRNA marker
siRNA (24nt)
h
miRNA marker(ng)
Transgenic SUPp
1.0 0.5 0.25 0.1 Col rMf rMf rMf
Probe
U6 & miR319 & miRNA marker
U6 RNA (101nt)
miR319 (21nt)
i
LFY and WUS
AAGAAT box
CCAAT boxes
BBS box for BLR
AG
MADS factors
1 bp
2999 bp
AGe
Mutant flowers (wMf & rMf)
% n
JM40 42 99
pR2509 2181 0 87
JM44 818 0 78
pR2088 908 0 93
JM43 1726 41 83
j
1726-bp endogenous AGe_rMfs
mCs(%)
100
50
0
CG
CHG
CHH
1726-bp transgenic AGe_rMfs
1273 (bp)
1500
2000
2500
2999
